# Supplementary material for: Further Validation Study of the Gender-Specific Binary Depression Screening Version (GIDS-15) and Investigation of Intervention Effects
Source: Behav Sci (Basel). 2025 Sep 14;15(9):1253. doi: 10.3390/bs15091253 (PMC12466554; doi:10.3390/bs15091253)
Supplement: Supplementary file 1 [file behavsci-15-01253-s001.zip › behavsci-3788030-supplementary.pdf]

Supplementary Materials

Table S1. Fixed effects regression coefficients with 95% confidence intervals of the mixed model with outcome GIDS-15 and participants as the random effect

Predictor	Estimate	95 % CI
IG	-0.13	-0.55; 0.29
Male sex	-0.26	-0.93; 0.40
T1	-0.64	-0.95; -0.32
T2	-0.71	-1.03; -0.39
IG x Male sex	0.13	-0.81; 1.07
IG x T1	-0.48	-0.95; -0.02
IG x T2	-0.55	-1.02; -0.07
Male sex x T1	0.29	-0.42; 0.99
Male sex x T2	0.69	-0.06; 1.42
IG x Male sex x T1	-0.40	-1.43; 0.64
IG x Male sex x T2	-1.32	-2.41; -0.24
Constant	3.46	3.17; 3.76

Note. CI = Confidence interval, IG = intervention group, T1 = measurement time 1, T2 = measurement time 2
